# Impact of the Recorded Audio and Video Feedback on Simulated Surgical Performance: A Randomized Controlled Trial

**DOI:** 10.7759/cureus.31621

**Published:** 2022-11-17

**Authors:** Shana Miles, Noah Rindos, Nicole Donnellan, Suketu Mansuria

**Affiliations:** 1 Obstetrics and Gynecology, Women's Health Clinic, Mike O'Callaghan Military Medical Center/Nellis Air Force Base, Las Vegas, USA; 2 Minimally Invasive Gynecologic Surgery Division, Allegheny Health Network, Pittsburgh, USA; 3 Division of Gynecologic Specialties, University of Pittsburgh Medical Center (UPMC) Magee-Womens Hospital, Pittsburgh, USA

**Keywords:** total laparoscopic hysterectomy, simulation in medical education, obgyn residency, laparoscopic technique, skills and simulation training

## Abstract

Introduction: Simulation and coaching have become increasingly important in laparoscopic skills acquisition. This study was designed to evaluate if access to the recorded audio and video feedback after a single proctored session improves the acquisition of laparoscopic suturing skills in obstetrics and gynecology (OB/GYN) residents.

Methods: Twenty OB/GYN residents received a single face-to-face coaching session on a laparoscopic vaginal cuff closure model. The session was recorded and residents were randomized to access either the video-only or the audio and video recording of the proctored session. The primary outcome measure was comparison of Global Operative Assessment of Laparoscopic Skills plus Vaginal Cuff Metrics (GOALS+) scores of the vaginal cuff closure prior to and following the proctored session.

Results: Only 30% of residents accessed the recorded sessions with junior residents most likely to access the recording. Baseline GOALS+ scores were significantly higher in senior residents (mean 21.7, SD 3.9) as compared to junior residents (mean 14.7, SD 3.2) (p<.001). While all learners' GOALS+ scores significantly improved after proctoring the intervention (p<.001), the senior residents continued to have significantly higher GOALS+ scores at the final assessment (mean 28.3, SD 4.2, p=.01) when compared to their junior residents (mean 24.0, SD 3.1).

Conclusion: Due to the low uptake of the review of recorded proctored sessions among OB/GYN residents across skill and year levels, we were unable to assess the effect of recorded audio and video feedback on resident performance. However, the intervention of a single proctored session of simulated laparoscopic vaginal cuff closure significantly improved resident performance as assessed with GOALS+ scores.

## Introduction

Recognizing the widespread practice of laparoscopy in the realm of benign gynecology, the American Board of Obstetrics and Gynecology has begun to require the completion of “Fundamentals of Laparoscopic Surgery” (FLS) for board certification [1]. Basic surgical simulators, such as those in FLS, have been shown to enhance live surgical performance in general surgery residents [2]. Surgical coaching can provide an additional benefit to this training. Simulation occupies a segment of the surgical curricula, while the mainstay of surgical training and performance relies on patient encounters. However, novice learners may be preoccupied with the procedure’s mechanics and not entirely receptive to the comprehensive feedback received “in the moment” intraoperatively. This cognitive load has been shown to affect performances of novices and experts in other task-based performances and has led to the evaluation of the effectiveness of post-operative debriefing and coaching [3-5].

While post-operative debriefing and video review with a skilled coach have been shown to improve performance, this requires significant time investment from the expert, a commodity not readily available at most institutions or practices [6]. There are currently no studies examining learners’ subsequent performance after audio and video review of their real-time (i.e., intraoperative or simulation) coaching feedback. Given the reduced surgical volume from duty hour restrictions and medical management in gynecologic surgery, augmenting the education from these limited patient encounters is essential to optimize surgical training programs/curricula. Recorded audio-video feedback from an expert coach’s intraoperative or simulated session could be integrated into surgical training curricula to maximize trainees’ surgical skills acquisition and coaches’ time and teaching.

This pilot study was designed to evaluate the performance of trainees during a simulated laparoscopic vaginal cuff closure when given access to the recorded proctored session with or without the audio feedback. We hypothesize that the learners who received access to the audio and video feedback would improve their performance more than those who received access to the video-only recorded sessions.

## Materials and methods

This study was a randomized controlled trial determined to be an exempt educational study by the University of Pittsburgh Institutional Review Board (#19080071). Study enrollment was open to obstetrics and gynecology residents at University of Pittsburgh Medical Center (UPMC) Magee-Womens Hospital, a 36-resident program at a large, tertiary, academic medical center. Twenty residents were enrolled and participated in the study between January 15, 2020, and July 28, 2020. In the setting of trainee COVID-19 restrictions, only 17 of 20 completed the final assessment. Due to uncertainty surrounding staffing and face-to-face simulation curricula, enrollment was halted, and interim analysis was performed.

At baseline, learners independently completed a recorded session of simulated vaginal cuff closure using three extracorporeal knots using the model described by King et al. and completed a survey [7]. They were then scheduled for a single session of private proctoring with an expert, fellowship-trained Minimally Invasive Gynecologic Surgery (MIGS) surgeon (S. Mansuria, or SM2). The expert provided contemporaneous verbal coaching and feedback during the session. This session was recorded, and learners were randomized to receive the video-only recording (control arm) or the audio and video recording of this proctored session. Learners were encouraged to review this recorded session prior to their final assessment. The learners were aware prior to the session that the proctored session was not graded and that none of the assessments would become part of their residency record. Learners were not aware of the primary study outcome measure being between types of recorded feedback and were only aware of their randomized groups at the time of accessing recording. The primary study investigator was automatically notified each time a recording was accessed.

Approximately one week after the proctored session, the learners returned for a final recorded session to independently complete the vaginal cuff closure task and another survey. The baseline and final suturing assessment videos were blinded by the primary investigator and then graded by two fellowship-trained MIGS surgeons (N. Donnellan, N. Rindos) using Global Operative Assessment of Laparoscopic Skills plus Vaginal Cuff Metrics (GOALS+). GOALS+ includes the GOALS proficiency domains of depth perception, bimanual dexterity, efficiency, tissue handling, and autonomy, and the vaginal cuff metrics of needle handling, knot tying and vaginal mucosa incorporation [7]. Autonomy was excluded from the assessment as learners were autonomous for both baseline and final sessions as the expert was present only during the proctored session. The seven remaining skills were graded using a 5-point Likert scale with a maximum score of 35. For each of these measures, higher scores correlated with higher levels of proficiency.

The primary outcome was a change in GOALS+ scores between the initial and final assessment between the audio-video and the video-only groups. Secondary study endpoints included change in GOALS+ scores by postgraduate year (PGY) level, number of times the proctored session recording was accessed, subjects’ performance assessment, and reported time in simulation lab between sessions. Extrapolating from Rindos et al. and King et al., we assumed GOALS+ scores would have a standard deviation of approximately 5 points and a pre/post correlation (within subjects) of 0.5 [6,7]. Power analysis was performed to detect 5-point differences between intervention groups with 80% power and 5% significant level, requiring 16 learners in each group. Data analysis was performed using Stata 15 (StataCorp LLC, College Station, TX). Frequency (n) and percentage (%) were used to describe baseline demographics with t-tests and one-way analysis of variance to evaluate training group differences. A paired t-test was used to evaluate differences in GOALS+ scores. Because of the limited sample size, PGY-1 and -2 were combined and PGY-3 and -4 were combined for analysis.

## Results

Twenty learners (six PGY-1, four PGY-2, five PGY-3, and five PGY-4) were enrolled in the study (Table 1). Due to the COVID-19 pandemic, recruitment was halted, and thus we were unable to power for the primary outcome. The pandemic led to unpredictable restrictions and availability of learners leading to three learners being unable to complete the final assessment and survey in the time permitted in the study protocol. Proctored sessions with the MIGS expert (SM2) ranged from 8:50 to 22:26 minutes, with an average of 16:32 minutes. Among all learners, only 30% accessed the recorded session as verified in the automated data management system; however, 53% learners reported accessing their personalized session. Five of the six learners who accessed the recorded coaching session were junior residents (three PGY-1, two PGY-2), with a single PGY-4 who accessed the recorded session. Of the three learners (one PGY-1, one PGY-2, one PGY-4) who were randomized to and accessed the recorded audio-video feedback, one accessed the recording twice, and the other two accessed it once. The three learners (two PGY-1, one PGY-2) who were randomized to and accessed the video-only, accessed the recording a single time each. Due to the small number of learners who accessed the recording, we could not determine if there were differences between the groups that received the video and audio recordings of their proctoring session and those that received the video-only recording.

**Table 1:** Demographics and surgical experience of participants FLS = Fundamentals of Laparoscopic Surgery; TLH = total laparoscopic hysterectomy; PGY = postgraduate year; M = male; F = female

	PGY-1	PGY-2	PGY-3	PGY-4
No. of participants	6	4	5	5
Gender	4 (F), 1 (M)	4 (F), 0 (M)	4 (F), 1 (M)	4 (F), 1 (M)
Mean age, years	26.8	27.5	29	29.8
Mean laparoscopic cases in the past 12 months (range)	2.3 (0-5)	11.8 (0-25)	46 (15-100)	65 (35-100)
Mean laparoscopic cases between initial/final session, primary surgeon (range)	1 (0-5)	0.33 (0-1)	3.3 (0-6)	4.6 (0-7)
Mean TLH, primary surgeon (range)	0 (0)	0 (0)	17.2 (0-30)	28 (10-50)
Mean laparoscopic vaginal cuff closure (range)	0 (0)	0.25 (0-1)	6.6 (0-20)	31 (20-45)
Mean laparoscopic vaginal cuff closure between baseline and final session (range)	0 (0)	0.33 (0-1)	2 (0-4)	2.8 (0-4)
FLS certified, n	0	0	2	5

**Figure 1:**
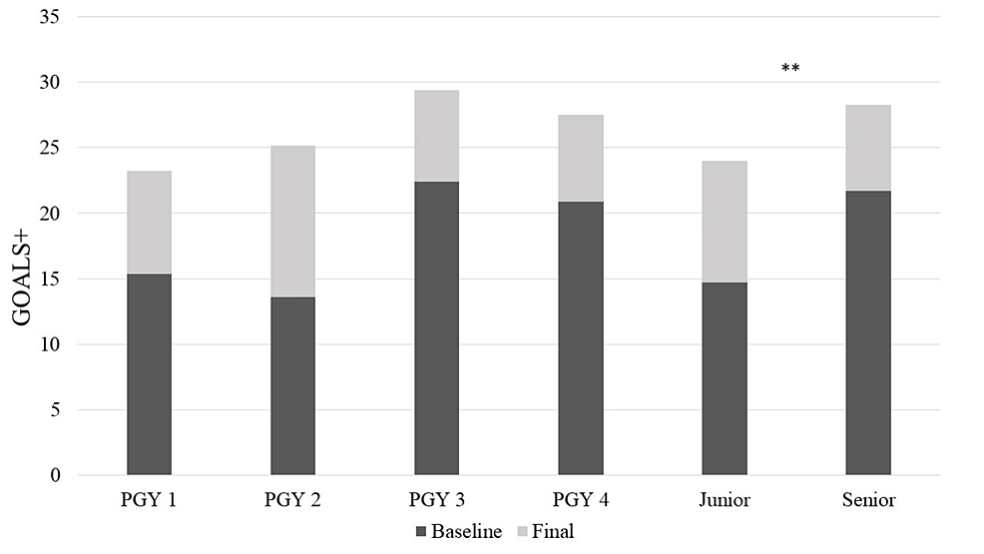
Mean baseline and final GOALS+ scores by the PGY level "Junior" shows the mean GOALS+ scores for all PGY-1 and PGY-2 residents and "Senior" is the mean GOALS+ scores for all PGY-3 and PGY-4 residents. **Baseline (p<.001) and final (p=.01) GOALS+ scores were significantly higher in senior residents as compared to junior residents. PGY = postgraduate year; GOALS+ = Global Operative Assessment of Laparoscopic Skills plus Vaginal Cuff Metrics

Inter-rater reliability scores for GOALS+ scores showed consensus (Cronbach’s alpha .94-.96, test scale .95; data not shown). Baseline GOALS+ scores differed between junior and senior residents with significantly higher scores in senior residents (mean 21.7, SD 3.9, p<.001) as compared to junior residents (mean 14.7, SD 3.2) (Figure 1). While all learners’ GOALS+ scores significantly improved after proctoring the intervention (p<.001), senior residents continued to have significantly higher GOALS+ scores at the final assessment (mean 28.3, SD 4.2, p=.01) when compared to their junior residents (mean 24.0, SD 3.1). Junior residents had a higher mean GOALS+ improvement (mean 8.9, SD 4.2) as compared to senior residents (mean 6.3, SD 2.9); however, this did not reach significance (p=.07).

In the interval between the initial session and the final session, learners reported an average of 33 minutes spent in the simulation lab. This was not evenly distributed among PGY levels, with PGY-1 averaging 60 minutes, PGY-2 37 minutes, PGY-3 38 minutes, and no PGY-4 reporting time in the simulation lab. Intraoperative experience in the interval between the baseline and final session varied among PGY levels with no vaginal cuff closures reported in PGY-1, one of the three learners closing a single cuff in PGY-2, an average of two cuff closures in PGY-3, and an average of 2.8 cuff closures in the PGY-4 level. Learners reported no additional robotic or vaginal cuff closure experience during this interval, with a single learner completing an abdominal cuff closure. When asked to self-assess improvement in their laparoscopic vaginal cuff closure skills, learners averaged a 33% improvement (PGY-1, 7.5%; PGY-2, 100%, PGY-3, 27.5%; PGY-4, 0%).

Despite only 30% of residents accessing the recorded session, at their final assessment, 65% of learners reported being likely or extremely likely to utilize the recorded video-only feedback and 76% reporting they were likely or extremely likely to utilize the recorded audio-video feedback in the future; 2 of the 17 learners who completed the final study survey reported they were ‘not at all’ likely to utilize the recorded audio or video feedback in the future, and these residents also did not access their recorded session during this study.

## Discussion

While we were unable to assess our primary outcome due to learners’ lack of utilization of this curricular resource, we demonstrated that the addition of a single, proctored coaching session improved GOALS+ scores on simulated laparoscopic suturing in all of our learners regardless of baseline performance. There was a trend for greater improvement in GOALS+ performance in our junior learners. Learners with no additional laparoscopic experience or time in the simulation lab demonstrated improvement in final GOALS+ performance, suggesting this proctored intervention with an expert MIGS surgeon had an effect on performance.

A coached review of learner performance on this same laparoscopic vaginal cuff model has been shown to improve resident performance on this task [6]. In this previous study, learners were randomized to receive weekly coaching meetings reviewing recorded performance in addition to the standard curriculum. When comparing results, our junior residents achieved similar final GOALS+ scores after a single proctored intervention (mean 24.0, SD 3.1) as compared to their reported performance after two weeks of the video-coaching-enhanced curriculum (mean 20.75, SD 3.1). The personalized coaching, whether using video coaching or “in real time”, appears to offer comparable results in learner performance on this vaginal cuff simulation model. This has significant implications considering barriers learners face with intraoperative skill acquisition due to the interplay of work hour restrictions, gynecologic surgical volume, patient safety, and the current COVID-19 pandemic. Identifying specific skills and implementing targeted skills interventions for practice and review outside of the high-stakes operating room may allow learners to maximize their limited time in the operating room. While this study did not evaluate intraoperative skill transfer, intraoperative skill performance has been correlated with FLS performance in general surgery residents [8]. Implementing targeted proctored or coached sessions at defined points in a resident’s training or continuing these modalities post-residency may be imperative for the development and maintenance of laparoscopic skills.

This study was initially designed to assess whether an independent review of audio and video feedback given during a performance-based task would enhance performance with no additional intervention needed by the instructor. We had hoped that recording intraoperative audio and video feedback could be a form of coaching that does not require any additional work, effort, or time by the coach outside of the operating room. Unfortunately, the majority of our learners did not access this resource. The learners commented they had "no time" allocated to access the recordings and that it was "not a requirement". Junior residents were most likely to access this recorded session with only a single senior resident accessing the recording. These junior learners with less operative experience, both prior to and during the study, augmented the experience with additional simulation time as compared to the senior learners. It is possible that if scheduled periods of self-assessment and review of performance-based feedback were incorporated into the resident duty day, they may have had additional motivation to review the recorded feedback and achieve higher performance levels. However, even without the review of these sessions, the specific feedback received during the sessions fine-tuning specific aspects of the skill, such as needle load, ergonomics, tension required for extracorporeal knot tying, improved learner performance. All learners, regardless of their baseline performance, improved on their final GOALS+ assessment following this single proctored session.

The strength of this study was the randomized controlled design and inclusion of learners across the PGY spectrum. A limitation of this study was the lack of structured time allocated in learners’ schedules to access the recorded session. In addition, the study was not designed to evaluate persistence of these skills over time or skill transfer to the intraoperative environment. The pandemic also impacted recruitment and ability to power our primary outcome, altering our ability to continue these assessments and coaching sessions. Three learners were unable to complete the final assessment in the time defined by our study protocol. The consistency of a standard vaginal cuff model to assess learner performance as compared to the anatomical variation and range of difficulty experienced intraoperatively assisted with learner performance in this study. While using a single coach (SM2) may limit the generalizability of this data, it controlled for more nuanced aspects of coaching and feedback unable to be assessed in this study protocol. The coach did not have a standardized checklist but was well versed in GOALS+ and provided feedback in real time during the sessions. Future studies could also examine the role of remote-proctoring for those in lower resource settings or low-volume practice. Skill transfer to intraoperative performance following simulation-based proctoring is another crucial next step when evaluating the role of simulation in the surgical education of gynecology residents.

## Conclusions

In conclusion, for residents to continue being safely trained to perform laparoscopic procedures, simulation must take a more important place in surgical curricula. With simulation supplementing practice-based skill learning, it will require additional time from skilled coaches in its current form. This study demonstrates that while learners are unlikely to access the supplemental recorded feedback from a proctored session of their own volition, 1:1 skill-based proctoring enhances skill acquisition of gynecologic residents. Both of these findings have significant implications for curriculum interventions. This study also highlights the untapped potential of utilizing surgical coaches that is particularly prescient for our military surgeons who are often faced with developing and maintaining their surgical skills in challenging settings both stateside and overseas.
